# A survey of individual preference for colorectal cancer screening technique

**DOI:** 10.1186/1471-2407-4-76

**Published:** 2004-11-08

**Authors:** Richard L Nelson, Alan Schwartz

**Affiliations:** 1 Department of Surgery, University of Illinois College of Medicine at Chicago, Chicago, Illinois 60612 USA; 2 Department of Medical Education, University of Illinois College of Medicine at Chicago, Chicago, Illinois 60612 USA

## Abstract

**Background:**

Due to the low participation in colorectal cancer screening, public preference for colorectal cancer screening modality was determined.

**Methods:**

A cross-sectional survey was performed of healthy ambulatory adults in a pediatrics primary care office and neighboring church. Overall preference was ranked for each of four colorectal cancer screening modalities: Faecal Occult Blood, Fiberoptic Sigmoidoscopy, Barium Enema and Colonoscopy. Four additional domains of preference also were ranked: suspected discomfort, embarrassment, inconvenience and danger of each exam.

**Results:**

80 surveys were analyzed, 57 of which were received from participants who had experienced none of the screening tests. Fecal Occult Blood Testing is significantly preferred over each other screening modality in overall preference and every domain of preference, among all subjects and those who had experienced none of the tests.

**Conclusions:**

Efforts to increase public participation in colorectal cancer screening may be more effective if undertaken in the context of public perceptions of screening choices.

## Background

Screening for colorectal cancer lessens the risk of dying from that disease [1]. Knowledge of this fact has not solved all the problems related to screening. The optimal modality of screening is still the subject of debate [1-3]. More problematic is the very low participation of the general public in recommended screening [4]. In contrast to breast cancer screening, in which the Healthy People 2000 Goal of the U.S. National Institutes of Health was surpassed, at 64% participation by women over 40 years of age, only 20% of Americans over age 50 had fecal occult blood testing within the past year (This is the best estimate of actual screening, rather than diagnostic endeavors for symptoms for which endoscopy or radiologic imaging might be done.), and 34% had a sigmoidoscopy within the past 5 years [5,6] Even if screening is appropriately performed, it is far from certain that a positive screen will be followed by appropriate diagnostic testing, as has been shown in follow-up surveys of fecal occult blood testing [7].

Most publication concerning colorectal cancer screening relates to the choice of screening modality; discussing accuracy, efficacy and cost, since the most inexpensive technique, faecal occult blood testing, is inaccurate in the detection of colorectal neoplasia, though effective in significantly diminishing disease specific mortality [8], and the most accurate technique, colonoscopy, is expensive and not without danger [3]. The choice is not an easy one for clinicians, much less patients or the asymptomatic public. Therein may lie one of the problems with public participation in screening. Unlike cancers of the breast, cervix, prostate or lung, where a single screening modality dominates current recommendations for each, there are four different and relatively independent screening tests for colorectal cancer that are currently recommended by the American Cancer Society, National Cancer Institute and United States Preventative Services Task Force: faecal occult blood test (FOBT), fiberoptic sigmoidoscopy (FS), barium enema (BE), and colonoscopy (C) [1]. The absence of a single recommendation may lead from indecision to inaction on the part of clinicians or patients.

However the greatest problem related to screening remains the low level of participation by those for whom it is intended: asymptomatic individuals over the age of 50 years with no specific risk factors for colorectal cancer, i.e., no past history of colorectal polyps, cancer, rectal bleeding, colitis, change in bowel habits, iron deficiency anemia, weight loss or a close family member with colorectal cancer. We agree with Dr. Woolf [2], that strategies to improve public compliance with recommended colorectal cancer screening might be more effective if they include an awareness of what the public thinks about the tests being recommended. Previous studies have not surveyed asymptomatic participants' preference over the whole range of screening choices, focusing instead on symptomatic patients undergoing diagnostic evaluation such as colonoscopy and barium enema [9-11] or patients ailing from extracolonic diseases whose motivation for screening might be very different than the healthy population for whom screening is intended [12-18]. Among these latter studies there has been a general preference noted for FOBT (table 1).

We have in this report chosen to focus our survey differently and uniquely; first to inform healthy, ambulatory and younger people, and not ailing patients, concerning only the preparation and conduct of each screening test. Secondly, in order to determine how their perceptions of the conduct of each test might affect their participation, participants were then asked to rank not just their overall preference based upon the preparation and conduct of the tests alone, but four other domains of preference for each screening modality: perceived physical discomfort, inconvenience, embarrassment and danger. Test accuracy was not included in the preamble on test performance, first, because we wanted to isolate perceptions of the physical conduct of the screening test, and second, because test accuracy has been part of many of the previous surveys, often presented with considerable bias. Randomized trials of decision aids have also shown that description of a test's ability to detect colorectal cancer has not been successful in increasing participation in screening [15-17]. Lastly, despite the current enthusiasm for screening colonoscopy by organizations that do colonoscopy as the complete screening test [19], as mentioned above, the choice of screening modality is still regarded as controversial.

## Methods

Participants were a convenience sample of parents or grandparents of children visiting a general pediatrics office (usually for well child visits or minor ailments), personnel working in that office, or parishioners attending a church social gathering, all aged 18 and over. An introductory letter described the purpose of the survey. This was followed by a brief description of the preparation and performance of each commonly used screening test for colorectal neoplasia: faecal occult blood testing (FOBT), fiberoptic sigmoidoscopy (FS), air contrast barium enema (BE) and total colonoscopy (C). The relative accuracy of each exam was not discussed. Six questions followed. The first asked the participant to rank each test in order of overall preference. The second asked the participant to rank each test according to how much that test might cause physical discomfort, the third, inconvenience; the fourth embarrassment, the fifth, the relative danger of the exam. The sixth question asked participants which of the four tests they had previously experienced, along with their gender and age. No further symptom or medical history was obtained and surveys were only numbered consecutively with no personal identifiers. (see appendix for letter and survey)

Based upon a related survey concerning subject preference for tests of colonic inflammation [20], a sample size of 50 individuals was estimated. Eighty four questionnaires were distributed in order to assure receipt of an adequate number of usable responses from individuals who had experienced none of the screening tests. The questionnaire is shown in the Appendix.

### Analyses

Data were analyzed using SPSS 11.0. Analyses focused on comparisons between ranks assigned each test on preference and the other assessed attributes, and included Friedman's test for ranks (to test the hypothesis that ranks differed for different tests) and the Wilcoxon signed-ranks test (to test the hypothesis that pairs of tests were differently ranked.) We also considered whether those rank orders might differ between participants who have and have not received any of these tests, and how gender and age affected preferences.

## Results

80 of 84 surveys were available for analysis; twenty nine from men and 51 from women. The mean age of the participants was 38.3 years (range 18 – 54 years; St. Dev. 8.19 years; median 40 years). Eight subjects had previously had a colonoscopy, five a barium enema, seven a sigmoidoscopy and 17 had stool collected for various reasons. Fifty seven subjects had experienced none of the screening tests. The mean rankings for preference among the entire sample are presented in Table 2 and among only those individuals who had experienced none of the tests are presented in the Table 3, score "1" being the most preferred and "4" the least. In each case, mean rankings were found to vary by test (Friedman's test, 3 df), and FOBT was significantly preferred over the second-ranked test (FS) by Wilcoxon signed-ranks test.

Median scores were determined for each domain for both the whole survey group and the naive subgroup. For each domain and in each group the results were the same, with ranks of 4,3,2 &1 for C, BE, FS and FOBT respectively, 1 being most preferred, except for embarrassment in both groups in which C and BE each had a median rank 3.

The results hold up for each gender subgroup in all cases except that men didn't consider FOBT significantly less inconvenient than FS. Age was not significantly correlated with ranking of FOBT (that is, the ranking given didn't change with age) by Spearman's rho. Rho values ranged from -0.10 to +0.16, none significant. Splitting the groups into ages 18–39 (n = 39) and 40+ (n = 40), the results are the same for both groups except that for those over 40, preference for FOBT vs. FS and inconvenience of FOBT vs. FS did not reach significance by two-tailed test (p = .079 and p = .057 respectively)

## Discussion

A recent review of colorectal cancer screening stated that, "At present there is no preferred CRC screening strategy"[1]. This presents the perspective of a group of impartial physicians. However from the perspective of those who should take part in CRC screening in the future, a clear preference for FOBT over each other screening modality is expressed in this survey. Each domain of preference similarly ranks FOBT as significantly most preferred.

Among previous surveys there are four randomized controlled trials of the use of decision aids that were designed with the intent of altering participation in screening. Three of these presented choices of screening modality or scenario to both intervention and control groups [15-17]. These studies therefore provided information of participant preference for specific screening modality, though again the participants, primary care *patients*, were quite different from the group reported herein. Only one of those reports offered all four of the screening modalities that we did in our study [16], the other two offering only a choice between FOBT and FS [15,16]. Nevertheless a uniform preference for FOBT was reported in these studies as well (Table 1). None of the test interventions were particularly effective in increasing participation in screening, an endpoint not assessed in our study. The fourth randomized trial randomized non-patients, relatives of gastroenterology patients, to be offered either FS or C and measured differential participation, which was equal in the two groups [20]. In the survey most similar to the present study, Pignone surveyed 146 patients in a general medicine clinic [18] and questioned participants after four sequential levels of information were given. Only two screening options were presented, FOBT & FS. Information included in sequence 1) the risk of colorectal cancer, 2) description of the conduct of the test, 3)accuracy of the tests, 4) cost. Previous screening participation was queried but not an exclusion. Less than 5% of those approached refused participation and no data were presented on the screening naïve participants in his sample. FOBT was preferred at each level of investigation, though both tests together were preferred after level 2 (Table 1). Participants were also asked for reasons for their preferences. The reasons most often given related to cost, ease of performance and being done alone.

Among some physicians there is a growing popularity for the use of definitive diagnostic testing as a screening tool, that is, colonoscopy [19]. Though expensive and not without danger, reimbursement for the test is declining and the procedure is getting safer. It has obvious theoretical advantages of offering precise diagnostic capabilities, through biopsy, for those with positive screens. Most important, colonoscopy has the best potential for cancer prevention by adenoma removal – which is not possible with any other test [22,23]. This, properly applied, might even result in cost savings in the global cost of caring for colorectal cancer.

But the public has to want to participate in this program and there is little evidence in this current survey and previous studies, especially those done in primary care settings [13-18], that this is likely. The concerns expressed herein about safety, embarrassment, inconvenience and discomfort all must be addressed in future efforts to increase screening participation. A potentially significant development related to these issues is that the principal disadvantage of FOBT, its inaccuracy in detecting colorectal neoplasia, might be overcome. Recently developed stool tests show an ability to diagnose cancer with much greater reliability [24]. Perhaps these gene based stool tests may establish the potential for adenoma discovery by non-invasive testing as well.

## Competing interests

The author(s) declare that they have no competing interests.

## Authors' contributions

RLN conceived of the study, designed the questionnaire and supervised its administration.

AS organized the domains of preference and performed the statistical analyses.

Both authors participated in the writing of the manuscript.

## Pre-publication history

The pre-publication history for this paper can be accessed here:



## Figures and Tables

**Table 1:** Surveys of screening preference for colorectal cancer

**First Author**	**Subjects Age Range**	**N =**	**Comparison**	**Preference**	**Comment**
Steine^9^	GI Patients 45–79 y	190	BE v C	BE	Post hoc
Durdey^10^	GI Patients 19–88 y	66	BE v C	C	Post hoc
VanNess^11^	GI Patients 20–84 y	189	BE v C	C	Post hoc
Elwood^20^	Relatives of GI Patients 45–70 y	232	FS v C	FS = C	RCT; Subjects offered FS or C and differential compliance measured
Dominitz^12^	VAOP 50–75 y	62	FS v C	FS	Time trade off measure Least for FS.
Frew^13^	PCOP >25 y	2294	FOBT v FS	FOBT	Willingness to pay primary endpoint. Preference also collected.
Leard^14^	PCOP 50–75 y	100	FOBT, FS, BE, C	C preferred FOBT more likely to be done.	Post hoc 93% previously screened
Dolan^17^	PCOP >50 y	96	FOBT, FS, BE, C	FOBT	DARCT
Pignone^15^	PCOP 50–75 y	227	FOBT v FS	FOBT	DARCT
Pignone^18^	PCOP 50–75 y	146	FOBT v FS	FOBT	4 levels of survey after varying quantities of information on colon cancer risk, conduct, test accuracy, cost.
Wolf^16^	PCOP >65 y	57	FOBT v FS	FOBT	DARCT
Nelson	Non-patients 18–54 y	80	FOBT, FS, BE, C	FOBT	

GI Patients; Gastroenterology patients

BE; Barium enema.

C; Colonoscopy

FS; Fiberoptic sigmoidoscopy

FOBT; Fecal Occult Blot Testing

Post hoc; Preference measured after undergoing one or more of the above screening tests.

RCT; Randomised Controlled Trial

VAOP; Veteran's administration hospital outpatients

PCOP; Primary care outpatients

DARCT; Randomised trial to investigate the effectiveness of decision aids in increasing screening participation

**Table 2:** Mean test ranks for each domain of preference of colorectal cancer screening test

	Dimensions				
Test Modality	Preference	Physical Discomfort	Inconvenience	Embarrassment	Danger

Colonoscopy	3.14	3.37	3.47	3.14	3.56
Barium Enema	2.87	3.09	2.97	3.12	3.56
Fiberoptic Sigmoidoscopy	2.38	2.46	2.04	2.40	2.32
Fecal Occult Blood Test	1.61	1.09	1.52	1.34	1.09
Friedman's test χ^2 ^(3 df)	62.7*	146.6*	110.9*	102.4*	162.8*
Wilcoxon signed-ranks Z (FOBT vs. FS)	4.1*	7.2*	3.2*	5.3*	7.5*
N =	77	79	79	78	79

Notes: Mean ranks for each test on each of the dimensions. Lower mean ranks refer to greater preference, and less discomfort, inconvenience, embarrassment, or danger. A * indicates test statistics that are significant at p < 0.05.

N = ; Less than 80 responses due to blank forms.

**Table 3:** Mean test ranks for each domain of preference of colorectal cancer screening test:: individuals who have experienced none of the tests

	Dimensions				
Test Modality	Preference	Physical Discomfort	Inconvenience	Embarrassment	Danger

Colonoscopy	3.13	3.42	3.56	3.07	3.57
Barium enema	2.83	3.11	2.98	3.15	3.05
Fiberoptic sigmoidoscopy	2.35	2.45	1.91	2.38	2.32
Faecal occult blood test	1.69	1.02	1.55	1.40	1.05
Friedman's test χ^2 ^(3 df)	38.7*	112.6*	86.6*	65.9*	118.4*
Wilcoxon signed-ranks Z (FOBT vs. FS)	3.2*	6.5*	2.0*	4.3*	6.6*
N =	54	55	55	54	55

Notes: Mean ranks for each test on each of the dimensions. Lower mean ranks refer to greater preference, and less discomfort, inconvenience, embarrassment, or danger. A * indicates test statistics that are significant at p < 0.05.

N = ; Less than 57 due to blank responses
